# Multidrug Idebenone/Naproxen Co‐loaded Aspasomes for Significant in vivo Anti‐inflammatory Activity

**DOI:** 10.1002/cmdc.202200067

**Published:** 2022-03-22

**Authors:** Nicola d'Avanzo, Maria Chiara Cristiano, Luisa Di Marzio, Maria Chiara Bruno, Donatella Paolino, Christian Celia, Massimo Fresta

**Affiliations:** ^1^ Department of Pharmacy University “G. d'Annunzio” of Chieti-Pescara Via dei Vestini n.31 66100 Chieti Italy; ^2^ Department of Experimental and Clinical Medicine University “Magna Græcia” of Catanzaro Campus Universitario-Germaneto Viale Europa 88100 Catanzaro Italy; ^3^ Department of Health Science University “Magna Græcia” of Catanzaro Campus Universitario-Germaneto Viale Europa 88100 Catanzaro Italy

**Keywords:** Aspasomes, topical drug delivery systems, multidrug nanocarriers and nanomedicine, anti-inflammatory properties, ascorbyl palmitate

## Abstract

The use of proper nanocarriers for dermal and transdermal delivery of anti‐inflammatory drugs recently gained several attentions in the scientific community because they pass intact and accumulate payloads in the deepest layers of skin tissue. Ascorbyl palmitate‐based vesicles (aspasomes) can be considered a promising nanocarrier for dermal and transdermal delivery due to their skin whitening properties and suitable delivery of payloads through the skin. The aim of this study was the synthesis of multidrug Idebenone/naproxen co‐loaded aspasomes for the development of an effective anti‐inflammatory nanomedicine. Aspasomes had suitable physicochemical properties and were safe *in vivo* if topically applied on human healthy volunteers. Idebenone/naproxen co‐loaded aspasomes demonstrated an increased therapeutic efficacy of payloads compared to the commercially available Naprosyn® gel, with a rapid decrease of chemical‐induced erythema on human volunteers. These promising results strongly suggested a potential application of Idebenone/naproxen multidrug aspasomes for the development of an effective skin anti‐inflammatory therapy.

## Graphical abstract

**Ascorbyl palmitate‐based nanocarriers** or aspasomes are a promising nanomedicine for thermal and transdermal delivery. The aim of this study was the synthesis of multidrug Idebenone/naproxen co‐loaded aspasomes to develop an effective anti‐inflammatory nanomedicine. Results demonstrated an increased therapeutic efficacy of payloads compared to the commercially available Naprosyn® gel and suggested a potential use of this nanocarrier for skin anti‐inflammatory therapy.

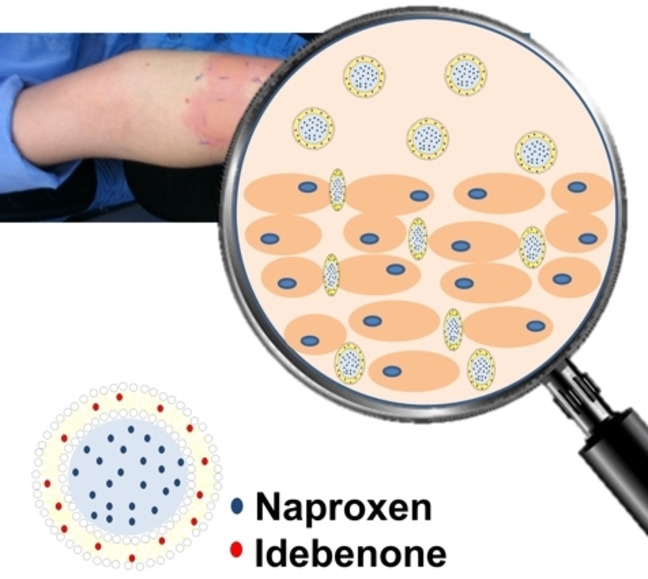

## Introduction

Epidermis is a complex and extended human tissue and provides a natural barrier which hampers bacterial systemic infections as well as the passage of toxic xenobiotics and molecules.^[1]^ However, epidermis anatomy and physiology decreased the dermal and transdermal efficacy of different drugs, thus significantly limiting the treatment of cutaneous diseases, such as local inflammation. To overcome these drawbacks, nanocarriers delivering bioactive molecules have been studied and their physicochemical properties were tailored to overcome stratum corneum‐epidermis and deep penetrate in the epidermis and derma. Currently, various drug delivery systems for topical applications have been widely studied based on their versatile and deformable properties.[^2^, ^3^, ^4^, ^5^] In this scenario, ascorbyl palmitate‐based nanocarriers, or aspasomes, had the best potentiality and promising properties for the treatment of cutaneous affections.[^6^, ^7^] Aspasomes are made up from ascorbyl palmitate, cholesterol and negatively charged lipids at different molar ratios.^[8]^ Ascorbyl palmitate, an ester derivative of ascorbic acid, has amphiphilic structure like surfactants and colloidal stabilizer. Moreover, its intrinsic antioxidant properties allow the synthesis of nanocarriers which results therapeutic *per se* and may potentiate the efficacy of payloads.^[9]^ These features make aspasomes proper nanocarriers for drug delivery in several inflammatory diseases that are strongly correlated to an increased production of reactive oxygen species (ROS).[^7^, ^10^] Based on this evidence, the aim of this work was the synthesis of aspasomes to co‐delivery anti‐inflammatory (naproxen) and antioxidant (idebenone) drugs for the topical treatment of cutaneous inflammation. Naproxen is a non‐steroidal anti‐inflammatory drug typically oral administrated which causes several gastrolesive effects.^[11]^ Unfortunately, naproxen, commercially available as sodium derivative, has not physicochemical properties which yield this drug suitable for dermal and transdermal delivery, thus limiting the opportunity to have significant topical anti‐inflammatory effects without any systemic toxicity. Idebenone is an ubiquinone derivative and potent antioxidant agent, which can inhibit lipid peroxidation and prevent skin aging and oxidative stress damages.^[12]^ The co‐delivery of naproxen and idebenone by aspasomes can increase the therapeutic efficacy of payloads and provide some advantages for multidrug therapy as previously reported for liposomes[^13^, ^14^, ^15^] and nanoparticles.[^16^, ^17^]

The lipid composition of aspasomes were firstly optimized and screened, as well as their manufacture to synthesize stable nanocarriers suitable for topical drug delivery. The resulting nanocarriers were physicochemical characterized and their long‐term stability were tested by Turbiscan Lab apparatus. Aspasomes with the best physicochemical and technological properties were selected to co‐deliver naproxen and idebonone and thus synthesize a performant multidrug carrier. Entrapment efficiency and release kinetic profile were studied *in vitro*, while the safety of nanocarriers were carried out both *in vitro* and *in vivo*. Finally, the anti‐inflammatory activity of naproxen and Idebenone co‐loaded aspasomes was tested *in vivo* on human volunteers after induction of harm chemical erythema. *In vivo* results demonstrated a significant anti‐inflammatory effect of resulting multidrug nanocarriers and provided a faster decrease of induced harm erythema than the commercial Naproxen‐gel medicine (Naprosyn®). These promising results suggested a potential anti‐inflammatory effect of this nanomedicine for cutaneous inflammation and its related pathologies and may provide an improvement of therapy currently market available.

## Results and Discussion

Aspasomes were tailored and optimized to have nanocarriers with the best physicochemical properties for dermal/transdermal delivery of payloads. The thin layer evaporation technique was used for the synthesis of nanocarriers as reported in the Experimental section, and they were obtained by combining lipids at different molar ratios (Table 1). Dimyristoyl phosphatidic acid (DMPA) or 1,2‐dimyristoyl‐sn‐glycero‐3‐phospho‐glycerol (DMPG) was always maintained at 10 % molar ratio, while L‐Ascorbic acid 6‐palmitate (6‐AAP) and Cholesterol (Chol) were changed to study the effect of different lipid molar ratio on physicochemical parameters of resulting vesicles. Dimyristoyl phosphate lipid, at 10 % molar ratio, was selected for the synthesis of aspasomes because this ratio stabilizes nanocarriers and avoids aggregation or modification of bilayer assembling and thermodynamic properties as previously reported.^[18]^ All the other lipids were selected to study the effect of negatively charged surface density, amino acid head derivatives, acylic pending chains of lipids and rigidity on the bilayer properties.^[19]^

**Table 1:** Lipid composition of aspasomes.

Formulation	6‐AAP [molar %]	Chol [molar %]	DMPA [molar %]	DMPG [molar %]
A1	65	25	10	–
A2	55	35	10	–
A3	45	45	10	–
A4	35	55	10	–
A5	25	65	10	–
A6	15	75	10	–
B1	65	25	–	10
B2	55	35	–	10
B3	45	45	–	10
B4	35	55	–	10
B5	25	65	–	10
B6	15	75	–	10

**Figure 1:**
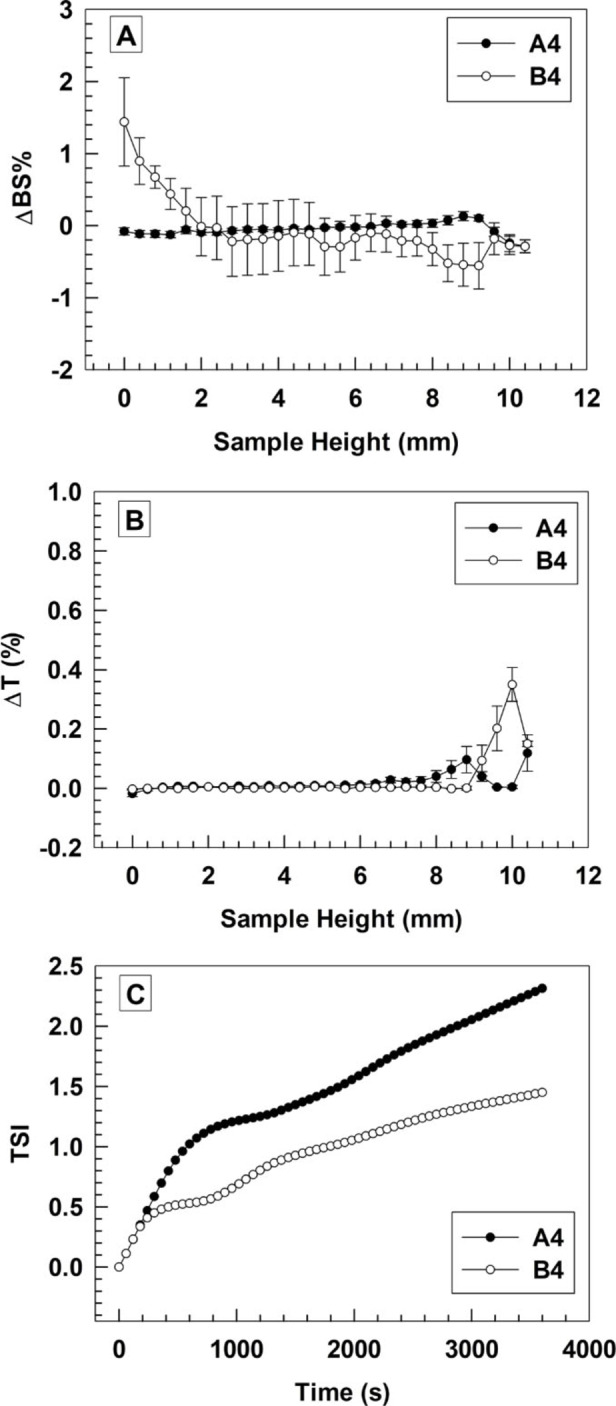
Turbiscan Lab analysis of formulations A4 and B4. Variation of backscattering profiles (A), variation of transmittance (B) and turbiscan stability index (C) were carried out as a function of sample height and incubation time (0–1 h). Analysis was performed at 25±0.5 °C and results are representative of three independent measurements ± standard deviation (S.D.).

The physical stability of nanocarriers as well as the prevention of flocculation, sedimentation, and aggregation phenomena after their synthesis or during storage at specific temperature and humidity for long‐time are mandatory for the topical application of colloidal drug delivery systems. These drawbacks can occur if colloidal suspensions products are stored for long‐time before administration or when nanocarriers are stressed many times with physical environmental stimuli like temperature variation, heating and UV‐Vis light. Turbiscan Lab apparatus is a solid and innovative equipment which tests the stability of emulsions, creams, foams, and colloidal nanoparticles and predicts their long‐term stability without disrupting samples and avoiding time‐consuming processes. The stability of colloidal nanoparticles as well as other formulations are monitored and related to the measurement and evaluation of infrared light transmitted and back‐scattered impulses as previously reported^[20]^ Physical theoretical parameters for delta backscattering (ΔBS%) and delta Transmission (ΔT%) profiles showed that colloidal suspensions are not stable (values over ±5 %) for samples with Chol molar % below or equal to 45 % (Figure S1 and Figure 1A and 1B). The stability of nanocarriers gradually increased by increasing the Chol amount up to 55 % molar ratio, while the use of Chol over 60 % caused a gradual decrease of physical stability of aspasomes. In fact, although A4, A5, A6, B4 and B5 formulations did not have ΔBS% and ΔT% values over the threshold of ±5 %, the lowest variation of ΔBS% and ΔT% were carried out for A4 and B4 formulations (Figure 1 and S1).

These results agreed with data previously reported and further endorsed the importance of Chol and DMPA/DMPG for the stabilization of aspasomes, because these amounts and combination of lipids can decrease and prevent the occurrence of mixed micelles and monolayer of 6‐AAP at the interface of aspasomes.[^8^, ^18^] The amount of Chol needed to stabilize Aspasomes is also similar to that used in niosomes, because 6‐AAP has a similar chemical structure of Span.^[21]^ Turbiscan stability index (TSI) of formulations A4 and B4 agreed with data obtained by ΔBS% and ΔT% analyses (Figure 1C), thus showing a kinetic profile which is like that previously reported for other stable colloidal suspensions.^[22]^

No significant differences were obtained for ΔBS% and ΔT% of formulations A 4 and B4, thus suggesting that DMPA and DMPG have a similar stabilizing effect on the aspasome bilayer although these lipids have different polar heads and charge intensity.

Average sizes and size distribution (polydispersity index or PDI) of aspasomes were carried out by using Dynamic Light Scattering (DLS). The resulting data showed that nanocarriers are broad distributed (PDI>0.3); this distribution may depend on the synthesis of mixed multilamellar and large unilamellar vesicles during the hydration stage.^[18]^

Results of DLS analysis for average sizes and PDI, agreed with data of Turbiscan Lab apparatus, and demonstrated that Chol at 55 % molar ratio (formulations A 4 and B4) stabilized aspasomes (Table 2).

**Table 2:** Physicochemical characterization of aspasomes.

Sample	Average size [nm]	Polydispersity index (PDI)	Zeta potential [mV]
A1	4720±743	0.925±0.109	−41.9±0.8
A2	3695±1356	0.999±0.003	−47.9±5.2
A3	1231±175	0.768±0.109	−45.5±4.0
A4	450±47	0.352±0.015	−46.1±1.4
A5	844±112	0.536±0.098	−53.3±2.5
A6	1183±1109	0.741±0.758	−55.3±1.3
B1	2232±49	0.885±0.091	−50.25±1.2
B2	1705±125	1.0	−50.21±2.3
B3	1469±281	0.998±0.005	−58.3±1.29
B4	389±37	0.439±0.098	−55.2±1.4
B5	1798±258	0.987 ±0.094	−60.25±1.4
B6	1985±10	0.401±0.059	−60.45±1.5

The zeta potential values of all samples were below −40 mV; this result demonstrates that aspasomes have a net negative surface charge which stabilizes nanocarriers and prevents their aggregation in the dispersing medium. In fact, a net negative or positive charge of colloidal nanoparticles below −30 mV or over +30 mV are indicative of stable colloidal nanocarriers which did not precipitate in the colloidal suspension. The high negative zeta potential value of aspasomes depended on the chemical properties of polar heads of ascorbic acid, making ascorbyl‐palmitate derivative as well as the high net negative charge of dimyristoyl phosphatidic acid (DMPA) or 1,2‐dimyristoyl‐sn‐glycero‐3‐phospho‐glycerol (DMPG) which are self‐assembled and make the lipid bilayer of nanocarriers. These high negative lipids and the additional negative charge of ascorbic acid strongly interacted in the lipid bilayer and thus make a complex supramolecular structure which has a net negative charge over −40 mV (Table 2). These results agreed with data previously reported elsewhere.^[18]^

Interestingly, aspasomes with DMPA had zeta potential values less negative than the respective colloidal nanocarriers with DMPG (Table 2). Differences of net negative surface charge of these samples were not completely clear; however, we speculated the hypothesis that the different steric hindrance of DMPA and DMPG polar head may modify the density of charge on the surface of aspasomes and thus the final net‐negative charge of nanocarriers. In fact, DMPA has a small polar head which can strongly interact with the head of 6‐AAP and Chol thus decreasing the net negative surface charge. Conversely, DMPG has one more glycerol molecule in the polar head than DMPA which can rearrange in the supramolecular structure of bilayer and put an additional hydroxyl group toward the external surface of bilayer, thus providing a more negative zeta potential value than DMPA aspasomes (Table 2).

Based on these results and their best physicochemical properties, the formulations A4 and B4 were selected for further studies. Selected aspasomes (formulations A4 and B4) were sonicated by using probe sonicator to decrease average sizes and have narrow size distributed nanocarriers. Sonication induces a mechanical disruption of external bilayer due to the input and diffusion of high frequency waves inside the aqueous colloidal suspension and this effect causes the overall decrease of average sizes.^[23]^ To test the impact of sonication on aspasomes, different amplitude (10, 30, 50, 70 % of maximal nominal power) was tested during the analysis and the resulting colloidal suspensions were visual analyzed to avoid the presence of particle metals released from the probe at the end of the process. Formulations A4 and B4 showed a significant decrease of average sizes after sonication and the reduction of particles increased by increasing the amplitude of sonication, due to the relative fragmentation of aspasomes because of the progressive increased mechanical stress induced on nanocarrier surface (Table S1). Similar results were obtained for size distribution which is narrow distributed by increasing the amplitude sonication up to 50 % with a PDI of maximum 0.17 (Table S1). The further increase of amplitude sonication at 70 % (son70 %) resulted in more increased values of PDI than aspasomes sonicated at amplitude of 50 % (son50 %) with maximum values of 0.168±0.024 vs 0.242±0.078, for formulation A4‐son50 % and A4‐son70 %, and 0.179±0.035 vs 0.289±0.104 for formulation B4‐son50 % and B4‐son70 %.

**Figure 2:**
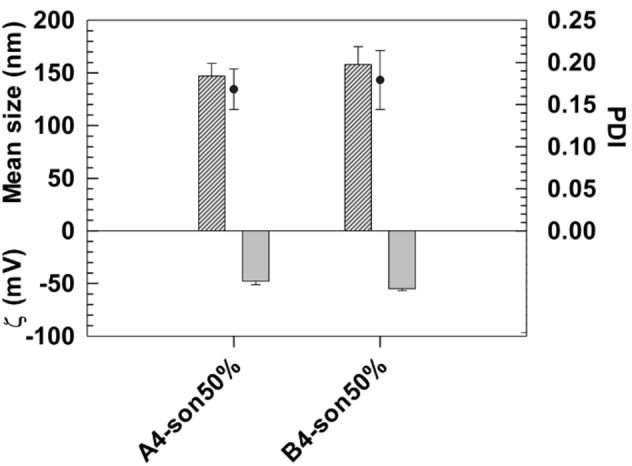
Physicochemical characterization of formulation A4 and B4 after probe sonication at 50 % of maximum amplitude power. Results are representative of three independent measurements±S.D.

Based on these results, amplitude sonication at 50 %, was selected for the synthesis of aspasomes loading therapeutic molecules and having the best physicochemical properties for a potential dermal application and further analysis. In fact, the PDI values of formulations A4 e B4 was below 0.2 and thus indicated a narrow size distribution of nanocarriers,[^24^, ^25^] while the resulting average sizes were 147±12 for formulation A4‐son50 %, and 158±17 nm for formulation B4‐son50 % (Figure 2).

No significant variations of zeta potential values were obtained for all samples after sonication, except for formulations sonicated at 70 % of maximum amplitude which showed a more net and significant negative surface charge of sonicated aspasomes than the same native nanocarriers (Table S1).

This modification of surface interface of aspasomes may depend on the excessive mechanical stress induced by sonication frequency at 70 % of amplitude, and the resulting rearrangement of supramolecular structure of aspasomes, which modify the spatial distribution of the exposed residuals functional groups on the surface of nanocarriers and the final shape of lipids making bilayer.^[26]^

The zeta potential values of aspasomes sonicated at different amplitude frequency were below −40 mV; this net negative surface charge prevents the aggregation of nanocarriers by electrostatic repulsion and improvement of dispersion in the suspension medium^[27]^ (Figure 2 and Table S1).

DLS analysis, collected from sonicated aspasomes, agreed with Turbiscan Lab analysis, and demonstrated that formulations A4‐son50 % and B4‐son50 % had the best profiles of ΔBS% and ΔT% with relative values below ±5% (data not shown).

Idebenone and naproxen were co‐loaded into A4‐son50 % and B4‐son50 % formulations to have a multidrug nanocarrier for potential application and cure of skin inflammation diseases. As previously discussed in the introduction section, idebenone is a synthetic analogue of Coenzyme Q10 with potent antioxidant properties.^[27]^ The radical scavenger properties of idebenone, which are similar to its parent drug Coenzyme Q10, makes this drug a potential candidate as adjuvant treatment of inflamed tissues, and prevents damages caused by the massive production of free radicals and the consequent generation of reactive oxygen species (ROS).^[28]^ Unfortunately, idebenone is poor water solubility and needs organic solvents, such as ethanol, to be solubilized before topical applications. Organic solvents, like ethanol, can significantly increase the local skin inflammation if applied topically in damaged and inflamed tissue.^[29]^ Moreover, Idebenone is quickly inactivated after exposition to UV‐Vis light with a resulting decrease of its antioxidant activity. To overcome these drawbacks and preserve the stability and activity of Idebenone, nanocarriers with suitable physicochemical properties have been synthesized.^[30]^ Moreover, Idebenone does not have suitable physicochemical and biopharmaceutical properties, like sodium naproxen, to pass by stratum corneum epidermis and reach the epidermis or derma where the anti‐inflammatory effects are required.^[31]^

For these reasons, Idebenone and naproxen were co‐loaded into aspasomes, thus providing a multidrug nanocarrier which further improves the anti‐inflammatory properties that aspasomes had *per se*.^[32]^ The loading of single drugs did not statistically modify average size and size distribution of empty nanocarriers, thus demonstrating that drugs are loaded in the aqueous compartment and bilayer based on their physicochemical properties and are well assembled with raw biomaterials making aspasomes (Table 3). A slight, but no statistically significant increase of average size (∼10 nm) was carried when sodium naproxen (as a single drug or in combination to idebenone) was loaded inside nanocarriers. (Table 3). This difference might depend on the increased osmotic pressure which occurred in the aqueous core of nanocarriers after the loading of naproxen, as previously reported for doxorubicin‐loaded liposomes.^[33]^ Statistically significant variations were obtained for zeta potential of A4‐son50 % and B4‐son50 % formulations when Idebenone was loaded as a single drug or in combination with naproxen. This effect might depend on the physical localization of Idebenone in the bilayer of aspasomes and its resulting interaction with other lipids of nanocarriers, which can modify the exposition of polar head groups for both DMPA and DMPG (Table 3). Conversely, the zeta potential of A4‐son50 % and B4‐son50 % formulations did not change significantly when naproxen is loaded as a single drug, thus endorsing the hypothesis that hydrophilic drug is overall entrapped inside the aqueous core of aspasomes and any drug molecules are adsorbed on their surface. Although aspasomes loading Idebenone had a significant decrease of zeta potential, the resulting net negative surface charge was below −40 mV for all drug(s) loaded nanovesicles. These values of zeta potential provide a suitable electrostatic repulsion between nanocarriers which can prevent aggregation and yield long‐term stability.

**Table 3:** Physicochemical characterization of Idebenone/naproxen loaded aspasomes.

Sample	Mean size [nm]	PDI	ζ [mV]	IDN E.E.%	NPX E.E.%
A4‐son50 %	147±12	0.168±0.024	−49.8±3.4	–	–
A4‐son50 % @IDN	145±9	0.159±0.087	−40.2±1.7*	49±2	–
A4‐son50 % @NPX	155±11	0.172±0.035	−51.0±2.2	–	65±6
A4‐son50 % @IDN+NPX	156±7	0.163±0.064	−41.3±3.1*	48±4	78±5
B4‐son50 %	158±17	0.179±0.035	−54.9±1.8	–	–
B4‐son50 % @IDN	155±13	0.181±0.051	−42.1±3.8*	51±4	–
B4‐son50 % @NPX	170±10	0.188±0.092	−52.8±4.6	–	36±3
B4‐son50 % @IDN+NPX	167±11	0.172±0.048	−44.6±1.7*	47±3	74±3

Abbreviations: ζ: zeta potential; IDN: idebenone; NPX: naproxen; @: embedding; son: probe sonicated; * *p*<0.05.

The physical stability of Idebenone/naproxen co‐loaded nanocarriers was further supported by Turbiscan Lab analysis, which showed values of ΔBS and ΔT variations below the threshold of ±5% (Figure S2) without showing any significant modifications of average size (kinetic diameter profile) of both A4‐son50 %@IDN+NPX and B4‐son50 %@IDN+NPX during the entire analysis (Figure S3). Results demonstrated that an average diameter of ∼170 nm was obtained for A4‐son50 %@IDN+NPX and B4‐son50 %@IDN+NPX nanocarriers. These results agreed data obtained by DLS analysis and further endorsed the solid physical stability of multidrug nanocarriers over the time.

The entrapment efficiency percentage (E.E.%) of both drugs was carried out by using proper calibration curves and no significant differences were obtained after quantification of payloads in the selected multidrug nanocarriers. In fact, our results demonstrated that A4‐son50 %@IDN+NPX (48±4, IDN and 78±5, NPX) and B4‐son50 %@IDN+NPX (47±4, IDN and 74±5, NPX) had a similar E.E.% for IDN and NPX when both drugs are co‐loaded in these nanoformulations (Table 3). The E.E.% of Idebenone was ca. 50 % for A4‐son50 %@IDN+NPX and B4‐son50 %@IDN+NPX formulations, while that of naproxen was 78±5 % and 74±3 % for formulation A4‐son50 %@IDN+NPX and B4‐son50 %@IDN+NPX, respectively (Table 3). Conversely, the E.E.% of aspasomes loading only naproxen was statistically significant different between A4‐son50 %@NPX and B4‐son50 %@NPX formulations (Table 3). E.E.% of naproxen was almost double for A4‐son50 %@NPX formulation compared to B4‐son50 %@NPX (65±6 % *vs* 36±3 %, respectively). These huge variations were unclear, but they may be associated to the different property of the polar head group of DMPA and DMPG. According to our previous speculation discussed in the manuscript, it is possible that the presence of an additional net negative charge of DMPA polar head as well as its smaller size than DMPG, promoted its interaction with other lipids of bilayer, thus increasing the overall rigidity nanocarriers and then providing a lower leakage of payload for A4‐son50 %@NPX formulation than B4‐son50 %@NPX formulation during the purification step. Conversely, no significant differences of E.E.% were obtained for aspasomes loading only Idebenone (Table 3), which had an E.E.% of ∼50 % for both A4‐son50 %@IDN and B4‐son50 %@IDN. Based on these results, the addiction of an extra lipophilic molecules, like Idebenone, may improve the stability of the bilayer and increase also the E.E.% of naproxen into the aqueous compartment. The “packaging effect” of Idebenone can then increase the amount of naproxen loaded in the multidrug nanocarriers, thus causing similar E.E.% for naproxen in A4‐son50 %@IDN+NPX and B4‐son50 %@IDN+NPX, although this effect was more evident for B4‐son50 %@IDN+NPX formulation (Table 3).

**Figure 3:**
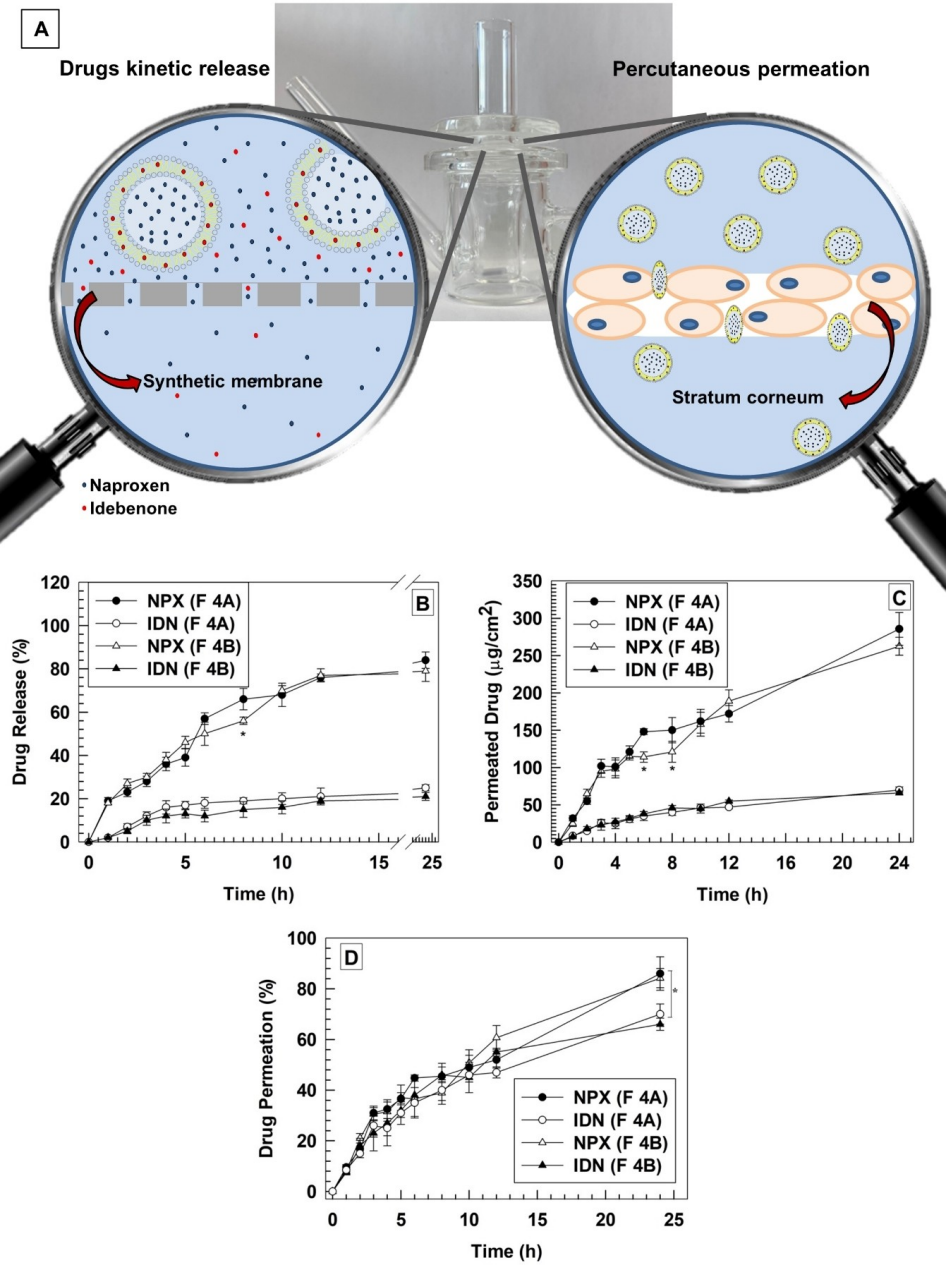
Kinetic release and percutaneous permeation of Idebenone and naproxen. (A) The upper part of figure is a schematic representation of drug release and percutaneous permeation of payloads from A4‐son50 %@IDN+NPX (F A4) and B4‐son50 %@IDN+NPX (F B4). The analysis was performed at 33±2 °C by using Franz diffusion cells equipped with synthetic cellulose and corneum and viable epidermis membrane for release (B) and permeation studies (C and D), respectively. Results are reported as the average of three independent experiments±S.D. * *p*<0.05.

The kinetic release profiles of Idebenone and naproxen were carried out by using Franz diffusion cells with a synthetic cellulose membrane (cut off 10,000 Da) between the donor and receptor compartments. A mixture of phosphate buffer saline (PBS) and ethanol (70 : 30 v/v) was used as a receptor medium to maintain the sink conditions during the experiment and avoid saturation of receptor medium for both hydrophilic and lipophilic drugs. The drug release percentage was quantified up to 24 hours, and the analysis of data showed a biphasic kinetic profile for naproxen with a rapid and continuous release up to 10 hours, followed by a pseudo‐steady state release up to 24 hours. A cumulative naproxen release of 83±4 % and 80±2 % for formulations A4‐son50 %@IDN+NPX and B4‐son50 %@IDN+NPX, respectively (Figure 3B). These results agreed with our previous hypothesis discussing the “packaging effect” of Idebenone which, similarly, to drug E.E.%, deleted differences of the release kinetic profile between A4‐son50 %@IDN+NPX and B4‐son50 %@IDN+NPX formulations (Figure 3B). Conversely, a slower and more sustained release profile was obtained for Idebenone, and a final cumulative drug release of ∼20 % was obtained after 24 hours of incubation for A4‐son50 %@IDN+NPX and B4‐son50 %@IDN+NPX formulations (Figure 3B).

These differences may depend on the physicochemical properties of Idebenone as well as its integration between acylic chains of lipids in the bilayer. The resulting release kinetic profiles may provide additional advantages for the potential application of aspasomes in inflamed skin. In fact, the fast release of naproxen can cause a rapid anti‐inflammatory effect, while the slow and sustained release of idebenone can prevent and/or decrease the synthesis of ROS. Moreover, we suppose that the presence of several mediators, such as blood proteins and enzymes, after *in vivo* application of nanocarriers may provide a further modification of the release kinetic of payloads due to the particle degradation inside the targeting tissue.

To better mimic *in vivo* conditions, Franz diffusion cells were also used to evaluate the permeation profile of aspasomes by human stratum corneum and viable epidermis (SCE) membrane. Results demonstrated that ∼250 μg/cm^2^ and ∼70 μg/cm^2^ of naproxen and idebenone, respectively, permeated by the SCE membrane after 24 hours of incubation (Figure 3C). The co‐delivery of Idebenone and naproxen caused a suitable permeation of both drugs through the human SCE membrane as a time dependent process with a pseudo‐zero order kinetic profile (Figure 3C). Although naproxen permeated by SCE membranes was almost four‐folder than Idebenone in 24 hours (Figure 3C), differences for the amount of permeated drugs depended on the higher E.E.% of naproxen than Idebenone (Table 3). In fact, data reported as percentage of permeated drug demonstrated that the differences between naproxen and Idebenone were significantly decreased and the permeated percentage was ∼70 % for Idebenone and ∼85 % for naproxen, after 24 hours (Figure 3D). These lower but still statistically significant differences can depend on specific physicochemical properties of selected drugs. In this attempt, we speculated that at specific incubation time naproxen may diffuse by the lipid bilayer, and it was then present in the donor compartment as free drug. In this scenario, it was possible that free molecules of naproxen were adsorbed on the surface of aspasomes close to the SCE membrane, and then permeated easier than the other molecules of drugs.

A second hypothesis, to explain this trend in the permeated drug percentage, was the slow and continuous release of Idebenone from aspasomes which occurred after the interaction between the SCE membranes and nanocarriers. Indeed, Idebenone is quite hydrophobic drug and its long interaction with the lipids of SCE membranes may cause its partial accumulation in the stratum corneous during the permeation process. The ∼15 % of difference for the permeated drug percentages of Idebenone and naproxen after 24 hours of incubation can depend on the combination of these two hypotheses. The lack of statistical differences between the permeation percentage of the two drugs in the early phase of incubation further supported our hypothesis, thus suggesting that differences depended on a time dependent cumulative effect. This slight modification did not modify the potential application of aspasomes as topical drug delivery systems. The permeation of aspasomes by the SCE membranes depended on the surfactant‐like property of 6‐AAP, which was like sorbitan derivatives, and its deformability and allowed the passage of nanocarriers by the tight junctions of the skin. These results suggested the potential use of aspasomes as dermal and/or transdermal drug delivery systems and were in agreement with results previously reported for transdermal delivery of azidothymidine.^[18]^

Biosafety of aspasomes was also studied to have safe and biocompatible nanocarriers and develop nanomedicine suitable for dermal and transdermal drug delivery in inflamed tissues.

*In vitro* toxicity of empty aspasomes was evaluated by using human keratinocytes NCTC 2544 cell lines at 24, 48 and 72 hours by the MTT test. Empty aspasomes were tested at the maximum lipid concentration equivalent to 100 μM and untreated cells were used as control.

**Figure 4:**
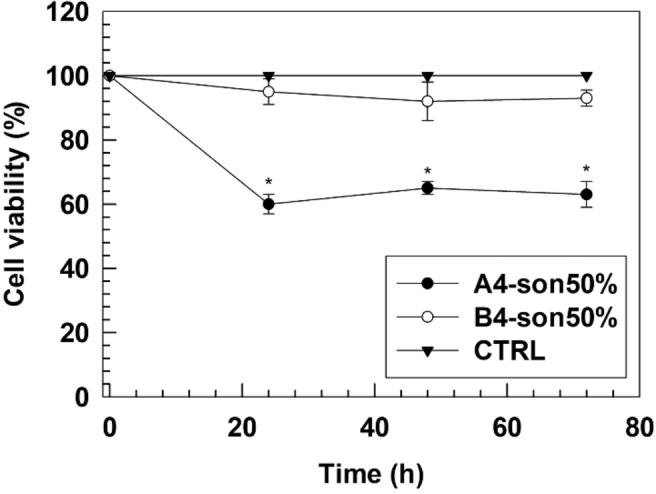
*In vitro* cytotoxic effect of empty nanovesicles on human keratinocytes NCTC 2544. Untreated cells were used as a control. Results are representative of three independent experiments±S.D. * *p*<0.05.

Results demonstrated different cytotoxicity for A4‐son50 % and B4‐son50 % formulations. A4‐son50 % caused a cell mortality of 40±3% after 24 hours of incubation and similar results were obtained up to 72 hours of incubation. Conversely, B4‐son50 % did not cause any significant modification of cell viability percentage which was ∼98 % up to 72 hours of incubation (Figure 4).

Differences of cell viability percentage between A4‐son50 % and B4‐son50 % formulations depended on their lipid composition and particularly the use of DMPA for A4‐son50 % formulation and DMPG for B4‐son50 % formulation. The additional molecule of glycerol in the polar head of DMPG may make this lipid more biocompatible with cells than DMPA. We can speculate that during the lipid exchange occurred between the cell membranes and the bilayer of aspasomes, the presence of DMPA can lead to the destabilization of cellular membrane and thus caused the resulting cytotoxic effect. This cytotoxicity was further increased

by static condition which is specific for *in vitro* tests, while it may change *in vivo* due to the sink conditions. This is the reason why we still used the formulation A4‐son50 % for the next studies and it was not discarded for *in vivo* analysis.

**Figure 5:**
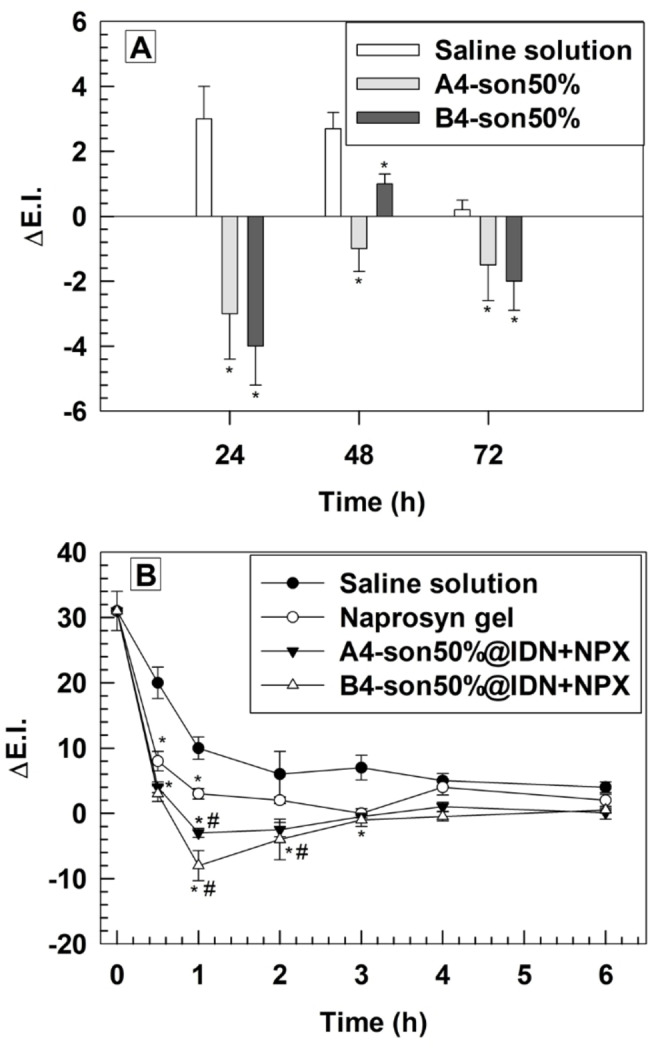
*In vivo* biosafety profiles of empty aspasomes (A) and anti‐inflammatory efficacy of Idebenone/naproxen co‐loaded aspasomes on human volunteers (B). Variations of erythematous index (Δ E.I.) were evaluated at different time points. Results are the mean of three independent experiments±S.D. (n=8). * *p*<0.05 (A4‐son50 %@IDN+NPX, B4‐son50 %@IDN+NPX and Naprosyn® *vs*. control); ^#^ *p*<0.05 (A4‐son50 %@IDN+NPX and B4‐son50 %@IDN+NPX *vs* Naprosyn®).

A4‐son50 % and B4‐son50 % formulations were studied *in vivo*, and their biosafety was tested on the harmful skin of human volunteers after written informed consent. The skin biosafety was studied by using a reflectance spectrophotometer (SP60 X‐Rite Incorporated) which evaluated the variation of Erythema Index (ΔE.I.) of harm's skin of human volunteers. After, ΔE.I. was measured at different time points (24, 48 and 72 hours) after the application of A4‐son50 % and B4‐son50 % (200 μL) formulations and the resulting data was compared to values obtained from controls which corresponded to the application of the same volume of a saline solution (NaCl 0.9 % p/V) on harm skin of human volunteers. Timing of incubation was selected according to the standard protocol of topical application for drug delivery. Moreover, treatment at short incubation times, such as 6 and 12 hours showed similar trend (data not shown). Formulation B4‐son50 % was safe *in vivo* and did not cause any cutaneous rush on the harm of human volunteers, thus agreeing with *in vitro* data. Despite formulation A4‐son50 % had a significant cytotoxic effect *in vitro*, any cutaneous rush occurred on the harm of human volunteers after its application on the skin (Figure 5A).

A4‐son50 % and B4‐son50 % formulations had a skin whitening effect after 24 hours of application. This effect was due to the presence of 6‐AAP in the lipid bilayer which maintained its intrinsic property of skin whitening agent after self‐assembling into the aspasomes.^[9]^ This property, combined to the capability to cross intact SCE membrane, made aspasomes suitable nanocarrier candidate for the treatment of inflamed skin, because they have a therapeutic activity *per se* and further increased the efficacy of payloads, by delivering and accumulating drugs inside the inner layers of skin.

Based on biosafety and suitable physicochemical properties, the anti‐inflammatory activity of A4‐son50 %@IDN+NPX and B4‐son50 %@IDN+NPX formulations were tested *in vivo* on human volunteers after written informed consent. A chemical erythema was induced on the specific skin areas (harm) of enrolled volunteers by using a methyl nicotinate solution (0.2 % w/v). The resulting chemical inflamed area was then treated with A4‐son50 %@IDN+NPX and B4‐son50 %@IDN+NPX formulations (200 μL) and the ΔE.I. values were evaluated by using reflectance spectrophotometer at fixed time points and up to 6 hours (30 min, 1, 2, 3, 4 and 6 hours). Saline solution (NaCl 0.9 % w/v) and commercial gel‐based naproxen (Naprosyn® 10 % w/v) were used as negative and positive controls, respectively. For Naprosyn®, the amount of gel was calculated to have equivalent concentration of naproxen loaded inside the aspasomes. The high ΔE.I. measured at time 0 indicated the successful induction of chemical erythema (Figure 5B). A4‐son50 %@IDN+NPX and B4‐son50 %@IDN+NPX formulations as well as Naprosyn® gel induced a statistical reduction of ΔE.I. compared to the negative control up to 3 hours of treatment. The maximum anti‐inflammatory effect of A4‐son50 %@IDN+NPX and B4‐son50 %@IDN+NPX formulations was obtained after 1 and 2 hours of incubation (Figure 5B). Indeed, at this time points both A4‐son50 %@IDN+NPX and B4‐son50 %@IDN+NPX formulations caused a statistically significant decrease of ΔE.I compared to the commercial naproxel gel (Figure 5B). The increased therapeutic efficacy of selected aspasomes depended on their capability to pass intact the SCE membranes and delivered payloads inside the targeting tissues as well as their intrinsic skin whitening properties. These properties suggest a potential application of aspasomes as multidrug nanocarriers for the treatment of skin inflammatory pathologies.

## Conclusion

In this study we synthesized ascorbyl palmitate‐based nanocarriers (aspasomes) for the effective co‐delivery of Idebenone and naproxen. We firstly optimized nanocarrier composition and synthetic process to have aspasomes with suitable properties for dermal and transdermal drug delivery. The best physicochemical properties were obtained for aspasomes made up by 6‐AAP, Chol and DMPA or DMPG at 35 : 55 : 10 lipid molar ratio after sonication at 50 % of maximum instrument amplitude output (formulations A4‐son50 % and B4‐son50 %). These nanocarriers had an average diameter below 160 nm, a narrow size distribution (PDI <0.2) and a net negative zeta potential (∼−50 mV). The co‐loading of idebenone and naproxen did not modify the physicochemical properties of aspasomes as well as their long‐term stability. Idebenone/naproxen co‐loaded aspasomes had an entrapment efficiency of ∼50 % and ∼75 % for Idebenone and naproxen, respectively, without any statistically significant differences between the selected nanocarriers. *In vitro* drug release studies demonstrated that a biphasic kinetic profile and a sustained and controlled drug release was carried out for both drugs up to 24 hours. The biosafety of empty aspasomes was carried out and endorsed both *in vitro* and *in vivo*, and aspasomes had a skin whitening effect due to the presence of 6‐AAP in their supramolecular structure. No significant differences were obtained between the two formulations for *in vivo* biosafety analysis, despite *in vitro* studies demonstrated a lower biosafety of A4‐son50 % than B4‐son50 %, with a human keratinocytes cell viability of 63±4% and 93±2.5 %, respectively, after 72 hours of incubation. The anti‐inflammatory activity of Idebenone/naproxen co‐loaded aspasomes was finally tested *in vivo* and demonstrated that they decreased the induced skin chemical erythema on human volunteers after 1 hour from topical application. Idebenone/naproxen co‐loaded aspasomes also increased the therapeutic efficacy of payloads compared to commercially available naproxen gel, particularly by 1 hour of topical application of formulations.

These promising results demonstrated a suitable use of Idebenone/naproxen co‐loaded aspasomes for the potential treatment of inflamed skin, thus showing a potential synergistic effect of payloads and ascorbyl palmitate self‐assembled inside the bilayer of nanocarriers. The improved efficacy of this nanomedicine compared to the commercial naproxen gel in addition to their biosafety profiles on human volunteers, strongly supported their topical application, to provide a more effective skin anti‐inflammatory therapy.

## Experimental Section

All materials and methods used in the study are reported in detail in the Supporting Information.

## Conflict of interest

The authors declare no conflict of interest.

1

## Supporting information

As a service to our authors and readers, this journal provides supporting information supplied by the authors. Such materials are peer reviewed and may be re‐organized for online delivery, but are not copy‐edited or typeset. Technical support issues arising from supporting information (other than missing files) should be addressed to the authors.

Supporting Information

## Data Availability

The data that support the findings of this study are available from the corresponding author upon reasonable request.
