# Supplementary material for: Multidrug Idebenone/Naproxen Co‐loaded Aspasomes for Significant in vivo Anti‐inflammatory Activity
Source: ChemMedChem. 2022 Mar 22;17(9):e202200067. doi: 10.1002/cmdc.202200067 (PMC9310947; doi:10.1002/cmdc.202200067)
Supplement: Supplementary file 1 — Supporting Information [file CMDC-17-0-s001.pdf]

ChemMedChem

Supporting Information

Multidrug Idebenone/Naproxen Co-loaded Aspasomes for Significant in vivo Anti-inflammatory Activity

Nicola d'Avanzo, Maria Chiara Cristiano, Luisa Di Marzio, Maria Chiara Bruno, Donatella Paolino,* Christian Celia,* and Massimo Fresta

Supporting information

Experimental Section

Materials

Cholesterol (Chol), ascorbic acid-6-palmitate, sodium naproxen, Trypan Bleu dye solution (0.4% v/v), 3-[4,5-dimethylthiazol-2-yl]-3,5-diphenyltetrazolium bromide (MTT) dye test (TLC purity ≥97.5%), sodium dimethyl sulfoxide (DMSO), phosphate buffer (PBS) solution and methyl nicotinate were purchased from Sigma-Aldrich (Milan, Italy). Dimyristoyl phosphatidylglycerol (DMPG) and dimyristoyl phosphatidic acid (DMPA) were obtained from Sigma Chemical. Co. (Steinheim, Germania). Idebenone was kindly provided by Takeda Italia Farmaceutici S.p.A (Rome, Italy) and was used without any further purification. Absolute ethanol, methanol and chloroform were purchased from Carlo Erba Reagenti (Milano, Italy). Double distilled pyrogen-free water was used throughout experimental studies. Spectra/Por cellulose membranes (cut-off 10,000), used for drug release tests, were provided by Spectrum Laboratories Inc. (Eindhoven, The Netherlands). Human keratinocytes (NCTC-2544) were provided by the Istituto Zooprofilattico of Modena and Reggio Emilia, Italy. DMEM (Dulbecco's Modified Eagle Medium) solutions, fetal bovine serum (FBS 10×), penicillin (100 UI/mL)-streptomycin (100 μg/mL) solution (1% v/v), and Trypsin/EDTA (1×) solution were obtained from GIBCO (Invitrogen Corporation, Giuliano Milanese (Mi), Italy). All other materials and solvents used in this study were of analytical grade.

Synthesis of aspasomes. Ascorbyl palmitate nanocarriers (aspasomes) were prepared by thin layer evaporation method as previously reported by Gopinath et al. with some modifications [1]. Different lipid molar ratios (**Table 1**) were studied. The resulting thin lipid films were hydrated with a PBS solution (10 mM, pH 7.4), warming at 55 °C and vigorous mixing (3 cycles). The suitable amount of buffer was used to obtain a final lipid concentration of 20 mg/mL. A probe sonicator (SONOPULS GM 70, Bandelin Electronic, Berlin, Germany) was used to optimize the physicochemical properties of resulting nanocarriers. Different sonication amplitudes were studied (10, 30, 50 and 70 % of maximum output). When required therapeutic nanocarriers were synthetized by dissolving Idebenone in the lipid mixture (5% w/w drug:lipid ratio), while naproxen was dissolved in the aqueous medium used for the hydration stage (10% w/w drug:lipid ratio). Un-entrapped drug was removed by size exclusion chromatography by using the Äkta Prime Plus (Amersham Biosciences, Uppsala, Sweden). Sephadex G-25 was used as stationary phase, while PBS solution (10 mM, pH 7.4) was used as mobile phase (the flow rate was set at 1 mL/min).

Physicochemical characterization of aspasomes. The prediction of the long-term stability of aspasomes were tested by using Turbiscan Lab® Expert (Formulaction, L'Union, France) as previously reported by Celia *et al* [2]. The analysis was performed at 25 °C and results carried out as variation of BS and T profiles as a function of incubation time and sample height. Average sizes, size distribution and zeta potential were carried out by a Zetasizer Nano ZS (Malvern Instruments Ltd., Worchestershire, United Kingdom) as previously reported [3].

Drug entrapment efficacy of aspasomes. Nanocarriers were centrifuged by an ultracentrifuge (Beckman, Fullerton, CA, USA) equipped with a fixed angle rotor Beckman mod. F1202 (95,000× g for 1 h at 4 °C) and the resulting pellets were dissolved with cold methanol. The amount of entrapped drugs was carried out by using a UV-Vis spectrophotometer (Perkin Elmer Lambda 20, Urbelingen Germany) at 281 nm and 331 nm, for Idebenone and naproxen, respectively. The drug entrapment efficiency was calculated following Equation (1):

$$E.E.\ \% = (D_{en}/D_{tot})*100 \qquad \text{(Eq.1)}$$

where, D_{en} and D_{tot} were the amount of entrapped drugs and added during the preparation procedure, respectively. An external calibration curve for both drugs was used during the analysis.

In vitro release kinetic profiles and percutaneous permeation studies. Release kinetic and percutaneous permeation studies were carried out by using Franz diffusion cells as previously reported with some modifications [4]. Synthetic cellulose (cut-off of 10,000 Da) and SCE membranes were used during the analysis [5]. All experiments with SCE membranes were performed in accordance with the Declaration of Helsinki, and the protocol was approved by the Research Ethics Committee of the University of Catanzaro "Magna Græcia" (Approval number: 390/2019). SCE membranes were obtained from the plastic reduction surgery of healthy adults (mean age 29 ± 4 years) and were isolated according to the published protocol [6]. The analysis was performed at 32.0 ± 0.5 °C. At fixed time points (30 min. and 1, 2, 3, 4, 5, 6, 8, 10, 12 and 24 hours), 500 µL of the receptor phase was withdrawn and replaced with fresh medium. The resulting samples were analyzed by UV-Vis spectrophotometer at 281 nm and 331 nm, for Idebenone and naproxen, respectively. An external calibration curve for both drugs was used during the analysis.

In vitro biosafety studies. Human keratinocytes cells (NCTC 2544) were used to evaluate *in vitro* biosafety of empty nanocarriers. The MTT test was used to investigate potential intrinsic cytotoxicity of nanocarriers. Cells were seeded in 96-well culture dishes (5×10^3 cells/0.2 mL) and after overnight incubation were treated with formulations 4A-son50% and 4B-son50% at the final lipid concentration of 100 µM. The cytotoxic effect of both samples was tested after 24, 48 and 72 hours of incubation. The formed formazan crystal was dissolved and the absorbance of the various samples was analyzed using an ELISA microplate reader (BIO RAD, xMarkTM Microplate Absorbance Spectrophotometer) at Abs 540 nm and 690 nm. The percentage of cell viability was calculated according to the following Equation (2):

$$\text{Cell Viability} = (Abs_T/Abs_C)*100 \qquad \text{(Eq. 2)}$$

where, Abs_T is the absorbance of treated cells and Abs_C is the absorbance of untreated cells (negative control).

In vivo skin tolerability of empty aspasomes. Reflectance spectrophotometer was used on human healthy volunteers to evaluate biosafety of optimized aspasomes. This non-invasive technique is based on the use of reflectance spectrophotometer SP60 (X-Rite Incorporated, USA) for monitoring the erythema index (EI). For these studies, eight healthy human volunteers (mean age 26 ± 4 years) were enrolled after the signature of informed consensus, and the approval of experimental protocol. All details of the protocol were provided and explained before starting the study which was carried out in accordance with the Declaration of Helsinki, and the protocol was approved by the Research Ethics Committee of the University of Catanzaro "Magna Græcia" (Approval number: 392/2019). Healthy human volunteers were housed in the day surgery room 30 for min. before the experiments at room temperature (22 ± 2 °C and 40–50% room humidity). The analysis were provided as previously described with slight variations [6]. *In vivo* skin tolerability was evaluated after 24, 48 and 72 hours and was calculated according to the following Equation (3):

$$E.I.=100[\log 1/R560+1.5(\log 1/R540+\log 1/R580) - 2(\log 1/R510+\log 1/R610)] \qquad \text{(Eq. 3)}$$

where, 1/R is the inverse reflectance at a specific wavelength (510, 540, 560, 580, 610).

In vivo anti-inflammatory activity of multidrug aspasomes. Anti-inflammatory properties of Idebenone/naproxen co-loaded aspasomes were evaluated *in vivo* on eight healthy volunteers. Briefly, a chemical erythema was induced by methyl nicotinate solution (0.2% w/v) on specific skin area (1 cm^2). After the induction of cutaneous erythema, 200 µL of each formulation was applied to inflamed skin. At fixed time points (1, 2, 3, 4, 5, 6 hours), the variation of E.I. was evaluated. Saline solution (NaCl 0.9% w/v) and commercial 10% Naprosyn® gel were used as negative and positive controls, respectively.

Statistical analysis. Statistical significance was evaluated by using One-way Anova test. A value of $p < 0.05$ was considered statistically significant. This analysis was performed with Microsoft Excel® (Microsoft Corp., Redmond, WA) and GraphPad Prism.

Supplementary Results

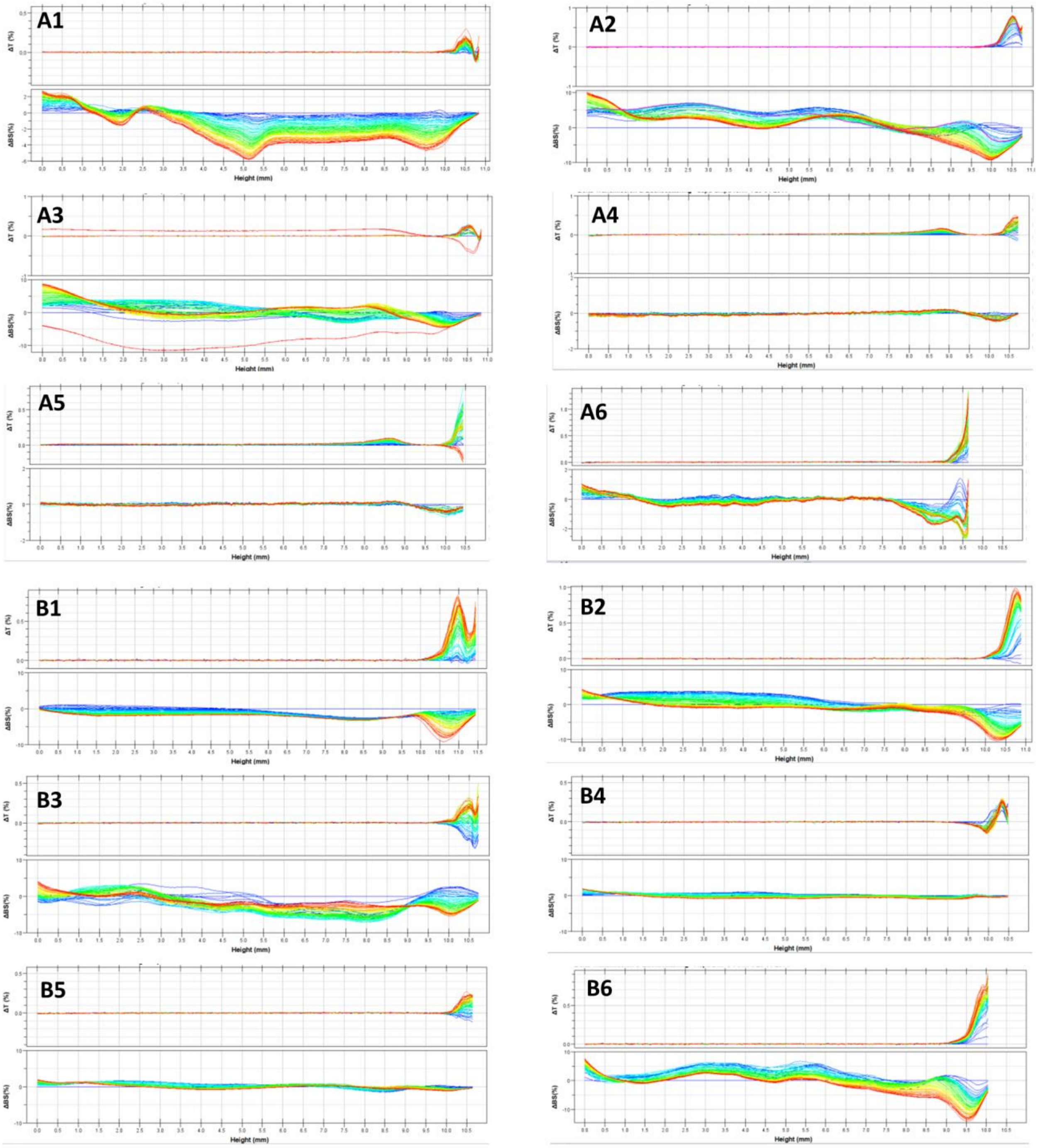

Figure S1. Variation of Backscattering (ΔBS%) and Transmission (ΔT%) profiles of aspasomes synthesized by using different lipid molar ratio (according to Table 1 included in the main text). Analysis was performed at 25 ± 0.5 °C and the resulting data are representative of three independent measurements.

Table S1. Physicochemical characterization of aspasomes after sonication at different amplitude output.

Formulation	Average size (nm)	PDI	Zeta Potential (mV)
**A4**	450 ± 47	0.352 ± 0.015	-46.1 ± 1.4
**A4-son 10%**	280 ± 38	0.289 ± 0.045	-50.5 ± 2.3
**A4-son 30%**	212 ± 25	0.225 ± 0.057	-51.4 ± 1.8
**A4-son 50%**	147 ± 12	0.168 ± 0.024	-49.8 ± 3.4
**A4-son 70%**	115 ± 28	0.242 ± 0.078	-66.8 ± 4.7
**B4**	389 ± 37	0.439 ± 0.098	-55.2 ± 1.4
**B4-son 10%**	354 ± 31	0.357 ± 0.095	-55.4 ± 2.7
**B4-son 30%**	275 ± 24	0.254 ± 0.087	-57.4 ± 3.8
**B4-son 50%**	158 ± 17	0.179 ± 0.035	-54.9 ± 1.8
**B4-son 70%**	124 ± 26	0.289 ± 0.104	-65.2 ± 4.1

Abbreviations: son: probe sonicated

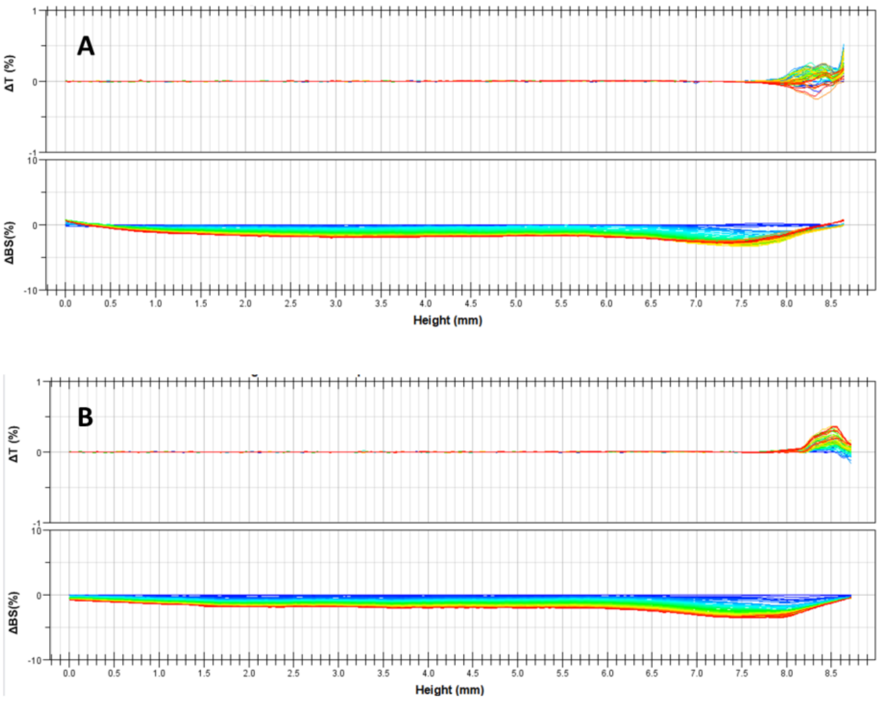

Figure S2. Variation of Backscattering (ΔBS%) and Transmission (ΔT%) profiles of A4-son50%@IDN+NPX and B4-son50%@IDN+NPX. Analysis was performed at 25 ± 0.5 °C as a function of sample height and incubation time (0-1 h). The resulting data are representative of three independent measurements.

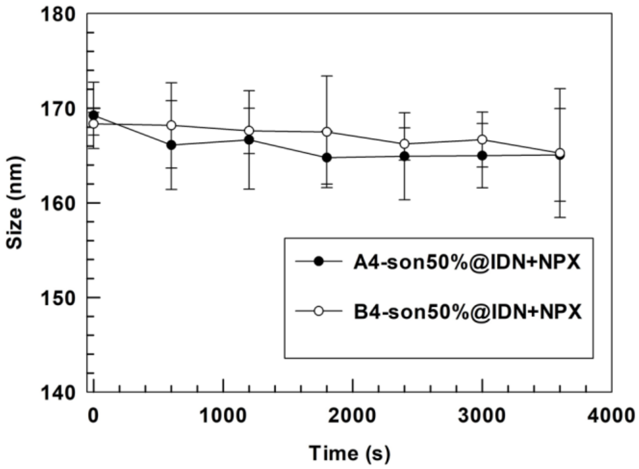

Figure S3. Diameter kinetic profiles of A4-son50%@IDN+NPX and B4-son 50%@IDN+NPX. Average diameter was carried out as a function of time (0-1 h). Results are the average of three different independent experiments ± S.D.

Supplementary references

[1] D. Gopinath, D. Ravi, B. Rao, S. Apte, D. Renuka, D. Rambhau, *Int. J. Pharm.* **2004**, *271*(1-2), 95-113.

[2] C. Celia, E. Trapasso, D. Cosco, D. Paolino, M. Fresta, *Colloids Surf. B.* **2009**, *72*(1), 155-160.

[3] M. Di Francesco, R. Primavera, S. Fiorito, M. C. Cristiano, V. A. Taddeo, F. Epifano, L. Di Marzio, S. Genovese, C. Celia, *Planta Med.* **2017**, *83*(05), 482-491.

[4] S. Nazir, M. U. A. Khan, W. S. Al-Arjan, S. I. Abd Razak, A. Javed, M. R. A. Kadir, *Arabian Journal of Chemistry* **2021**, *14*(5), 103120.

[5] D. A. Kuznetsova, L. A. Vasileva, G. A. Gaynanova, E. A. Vasilieva, O. A. Lenina, I. R. Nizameev, M. K. Kadirov, K. A. Petrov, L. Y. Zakharova, O. G. Sinyashin, *Int. J. Pharm.* **2021**, *605*, 120803.

[6] D. Paolino, G. Lucania, D. Mardente, F. Alhaique, M. Fresta, *J. Control. Release* **2005**, *106*(1-2), 99-110.
